# Solid-phase microextraction/gas chromatography–mass spectrometry method optimization for characterization of surface adsorption forces of nanoparticles

**DOI:** 10.1007/s00216-014-8078-z

**Published:** 2014-08-29

**Authors:** Enisa Omanovic-Miklicanin, Sandro Valzacchi, Catherine Simoneau, Douglas Gilliland, Francois Rossi

**Affiliations:** 1 Nanobioscience Unit, Institute for Health and Citizen Protection, European Commission-Joint Research Centre (JRC), Via Enrico Fermi, 2749, 21027 Ispra, VA Italy; 2 Chemical Assessment and Testing Unit, Institute for Health and Citizen Protection, European Commission-Joint Research Centre (JRC), Via Enrico Fermi, 2749, 21027 Ispra, VA Italy; 3 Chemistry Department, Faculty of Agriculture and Food Sciences, Zmaja od Bosne 8, 71 000 Sarajevo, Bosnia and Herzegovina

**Keywords:** Nanoparticles, Characterization, Interactions, Biomolecules

## Abstract

A complete characterization of the different physico-chemical properties of nanoparticles (NPs) is necessary for the evaluation of their impact on health and environment. Among these properties, the surface characterization of the nanomaterial is the least developed and in many cases limited to the measurement of surface composition and zetapotential. The biological surface adsorption index approach (BSAI) for characterization of surface adsorption properties of NPs has recently been introduced (Xia et al. Nat Nanotechnol 5:671–675, 2010; Xia et al. ACS Nano 5(11):9074–9081, 2011). The BSAI approach offers in principle the possibility to characterize the different interaction forces exerted between a NP's surface and an organic—and by extension biological—entity. The present work further develops the BSAI approach and optimizes a solid-phase microextraction gas chromatography–mass spectrometry (SPME/GC-MS) method which, as an outcome, gives a better-defined quantification of the adsorption properties on NPs. We investigated the various aspects of the SPME/GC-MS method, including kinetics of adsorption of probe compounds on SPME fiber, kinetic of adsorption of probe compounds on NP's surface, and optimization of NP's concentration. The optimized conditions were then tested on 33 probe compounds and on Au NPs (15 nm) and SiO_2_ NPs (50 nm). The procedure allowed the identification of three compounds adsorbed by silica NPs and nine compounds by Au NPs, with equilibrium times which varied between 30 min and 12 h. Adsorption coefficients of 4.66 ± 0.23 and 4.44 ± 0.26 were calculated for 1-methylnaphtalene and biphenyl, compared to literature values of 4.89 and 5.18, respectively. The results demonstrated that the detailed optimization of the SPME/GC-MS method under various conditions is a critical factor and a prerequisite to the application of the BSAI approach as a tool to characterize surface adsorption properties of NPs and therefore to draw any further conclusions on their potential impact on health.

**Graphical Abstract:**
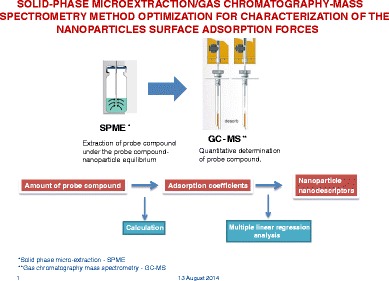
The basic principle of SPME/GC-MS method for characterization of nanoparticles surface adsorption forces

The basic principle of SPME/GC-MS method for characterization of nanoparticles surface adsorption forces

## Introduction

The rapid development of nanotechnological products raises concern on their possible adverse effects on health and environment. In fact, potential toxicity of NPs to humans is the object of a large debate and it is recognized that a consistent understanding and evaluation of the NPs behavior critically depends on the reliable and complete characterization of the NPs physical chemical properties. The knowledge of defined properties-toxicity relationships is a prerequisite for NPs evaluation. The physico-chemical properties to be evaluated are relatively well known and include for instance size distribution, composition, solubility, crystallinity, specific surface area, surface charge, etc. [1–5]. Despite the recognition that NPs surface properties are essential in determining the interaction of NPs with biological systems, relatively few properties are measured, and in most of the cases limited to specific surface area, zeta potential, and surface composition. These characteristics cover only limited number of the properties determining biological interactions [6, 7].

The biological surface adsorption index (BSAI) presents a novel approach for surface characterization of NPs in biological systems [8, 9]. It has been developed to identify and quantify the significant interaction forces that govern the adsorption properties of biomolecules (organic compounds, peptides, proteins, etc.) on NPs.

The BSAI approach consists of a quantification of the adsorption on the NP surface of different organic probe compounds with diverse structural properties, which senses the different interactions forces between the probe and the NPs surface. The calculation of adsorption coefficients is used to create a set of nanodescriptors by means of multiple linear regression analysis, which represent the contributions and relative strengths of molecular interactions (London dispersion forces–hydrophobic interactions, hydrogen bond acidity and basicity, dipolarity/polarizability, and lone-pair electrons) that exist between NPs and biomolecules. These nanodescriptors can then be used to develop pharmacokinetic and safety assessment models for NPs.

The contributions of each type of molecular interaction are experimentally determined by measuring the adsorption of the different probe compounds. Solid-phase microextraction–gas chromatography mass spectrometry (SPME/GC-MS) represents a simple and rapid technique for the quantification of organic compounds in different matrices based on their adsorption on different polymeric fibers and, with its continuous development, it has found applications in various fields of research [10–12]. Because of its ability to extract and quantify target compounds in aqueous solutions without modifying the solution chemistry, it has been successfully applied in studies on the adsorption of organic compounds on NPs [13]. In fact, SPME technique permits a selective extraction of the target organic compounds not adsorbed by the NPs directly into the reaction vessel, with a reduced effect on the interaction between NPs and probe compounds. In particular, this analytical approach can offer an advantage compared to other analytical techniques, since it does not require the removal of NPs before the analysis, with ultrafiltration or ultracentrifugation procedures, which could determine the release of the probe compounds adsorbed onto the surface of the NPs, thus affecting the result of the adsorption. On the other hand, because of the presence of NPs suspended in solution, the effects on the analytical results due to their possible adsorption onto the SPME fiber have to be considered.

In this study, we performed detailed optimization of the method for the quantification of the adsorption of probe compounds by NPs using SPME/GC-MS analysis. We optimized the conditions applied for the development of the interactions between the NPs and the probe compounds to permit their correct quantification. The interactions between NPs and SPME fiber were also investigated. Quantification of the adsorption coefficients represents a fundamental part in BSAI approach and the accuracy of adsorption coefficients calculation will determine the correct calculation of nanodescriptors. To our knowledge, this is the first time that all SPME/GC-MS method parameters were optimized. This should allow application of the method in different environments and different laboratory conditions which was not the case till now. The optimized method was tested for the measurement of the adsorption coefficients of organic probe compounds with diverse physico-chemical properties for Au NPs (15 nm) and SiO_2_ NPs (50 nm). Adsorption coefficients were correlated with solute descriptors by means of multiple linear regression analysis to obtain the nanodescriptors for each type of NPs. Although previous studies [8, 9] explained application of the BSAI approach in characterization of different NPs using SPME/GC-MS analysis of probe compounds, they lack a comprehensive evaluation and optimization of the analytical method.

## Experimental approach

### Materials and reagents

Au NPs (15 nm) and SiO_2_ NPs (50 nm) were synthesized as described in “Au NPs (15 nm) synthesis” and “SiO_2_NPs (50 nm) synthesis” sections.

All chemicals used in the study were of analytical grade and purchased from Sigma-Aldrich (Steinheim, Germany).

### Au NPs (15 nm) synthesis

Citrate-stabilized Au NPs of ∼15 nm in diameter were prepared in water by a modification of the Turkevich approach [14, 15]. Briefly, 5 mL of an aqueous solution of gold (III) chloride trihydrate (10 mM) were added to 95 mL of Milli-Q water in a 100-mL round bottom flask equipped with a magnetic stirrer and a Vigreux column. The mixture was heated rapidly (<1 min) to 97 °C using a microwave synthesis system at 150 W (Discover SP, CEM corporation) and then kept for 5 min. A solution of sodium citrate tribasic dihydrate (100 mM) was added under vigorous stirring and the reaction mixture was maintained at 97 °C for 20 min before the reaction vessel was rapidly cooled to 60 °C with compressed air. The resulting suspension was then allowed to cool to room temperature.

### SiO_2_ NPs (50 nm) synthesis

To obtain monodispersed SiO_2_ NPs in the range below 80 nm, it was necessary to adopt an alternative strategy based on the recent publications of Hartlen et al. [16]. In the method described, the NPs were produced in a growth medium of water containing arginine or lysine catalyst. The water/amino acid mixture was placed in a glass vessel and a smaller volume of an immiscible organic liquid (cyclohexane) added, and allowed to float above the aqueous phase. This biphase solution was then heated (if necessary) and allowed to stabilize at the desired reaction temperature (60 °C) with slow stirring of the aqueous layer. In the case where additional heating was supplied, this was done using a microwave system (Discover SP, CEM Corporation). Tetraethyl orthosilicate (TEOS) was then slowly added to the organic phase where it was preferentially dissolved. The system was then left under slow stirring for time periods of a few hours to several days. During this time, a slow diffusion was observed of TEOS from the upper organic layer into the aqueous phase where hydrolysis and condensation occurred with the nucleation and growth of small SiO_2_ NPs with narrow size distribution (24 ± 3 nm).

The microwave heating system could be used for maximum batch volumes of 100 mL; therefore, this volume was established as the standard volume for SiO_2_ NPs synthesis by the biphase method.

To regrow silica seeds to a desired size (50 nm), an appropriate portion of the seeds of small SiO_2_ NPs (24 nm) was taken and diluted with water. The final arginine concentration was in the range of 1 to 2 mM. Cyclohexane was added subsequently to ensure that the final volume ratio of total TEOS to cyclohexane remained at or slightly below 1:1. The mixture was then brought to 60 °C under constant stirring of ca. 300 rpm and a required amount of TEOS was added to the solution at once. Once TEOS was added, the reaction was allowed to proceed for 30 h while maintaining the stirring and constant temperature of 60 °C.

### Preparation of probe compounds solutions

Primary standards of probe compounds were prepared in methanol at concentrations that permitted the addition of a minimum volume of methanol (5 μL) in the solution used for adsorption experiments to minimize its effect on the capacity of the SPME fiber. The compounds tested represented a group of substances with chemical structures that have been widely used for quantitative structure–activity relationship (QSAR) models [17, 18].

### Sample preparation

The samples for SPME/GC-MS analysis were prepared by mixing 100 μL of NPs suspension, 95 μL of a phosphate buffer solution (50 mM), and 5 μL of methanol solutions of the probe compounds in 2-mL glass vials with a low-volume insert of 0.3 mL.

Control samples were prepared in the same type of vials by mixing 100 μL of sodium citrate tribasic dihydrate solution (100 mM) to match the composition of NPs suspension, 95 μL of a phosphate buffer solution (50 mM), and 5 μL of methanol solutions of the probe compounds. Adsorption experiments were carried out with probe compounds at the concentrations reported in Table 1.

**Table 1:** List of the probe compounds tested in the adsorption study with concentrations

	Probe compound	ng mL^−1^		Probe compound	ng mL^−1^
1	Chlorobenzene	19.8	18	4-Chloroanisole	41.4
2	Ethylbenzene	15.5	19	Phenethyl alcohol	240
3	p-Xylene	15.3	20	3-Methylbenzyl alcohol	237
4	Bromobenzene	26.4	21	4-ethylphenol	70.7
5	Propylbenzene	15.1	22	3,5-Dimethylphenol	70.7
6	4-Chlorotoluene	18.7	23	Ethyl benzoate	36.9
7	Phenol	71.1	24	Methyl 2-methyl benzoate	37.9
8	Benzonitrile	36.0	25	Naphtalene	17.7
9	4-Fluorophenol	70.7	26	3-Chlorophenol	85.3
10	Benzyl alcohol	24.6	27	4-Nitrotoluene	35.4
11	Iodobenzene	31.9	28	4-Chloroacetophenone	41.3
12	Acetophenone	72.8	29	3-Bromophenol	111
13	3-Methylphenol	73.1	30	1-Methylnaphtalene	16.9
14	Methylbenzoate	38.5	31	Biphenyl	17.8
15	2-Phenylphenol	75.5	32	Benzoylbiphenyl	17.5
16	Phenanthrene	80.0	33	Pyrene	25.0
17	Ethylnaphthalene	20.0			

### Quantification of the adsorption of probe compounds by SPME fiber

The SPME fiber used for the experimental was a Supelco StableFlex 65 μm (film thickness), 23 Ga (needle diameter), divinylbenzene/polydimethylsiloxane (DVB/PDMS) (Supelco, Bellefonte, PA, USA). A biphasic fiber was chosen to cover a wider range of analytes and the efficacy of DVB/PDMS fiber coating for the adsorption of the compounds considered in this experiment was already showed by previous studies [8, 9]. The optimization of the adsorption of probe compounds onto the SPME fiber was carried out on control samples (without nanoparticles) prepared as described in “Quantification of the adsorption of probe compounds by SPME fiber” section. The fiber in SPME analysis can adsorb sample compound from the gas phase in the vial (headspace sampling) or directly from the sample solution (liquid-phase sampling). Gas-phase sampling prevents a direct contact of fiber with liquid sample, reducing interferences from matrix components in the adsorption step. However, gas-phase sampling could led to a loss of the probe compounds through the headspace of the vial during their adsorption by NPs, determining incorrect results, in particular for compounds with long equilibrium time and low solubility. In this perspective, liquid-phase sampling performed directly into a vial without headspace can represent a suitable solution and it allows a complete automation of the analytical procedure. In the present study, the analysis was carried out with liquid sampling technique using glass vials for autosampler filled with liquid sample to a level that permitted the immersion of the fiber only and not the syringe needle. The temperature of the sample during the SPME adsorption was set at 30 °C to avoid the effect of excessive temperatures during the following tests on the adsorption by nanoparticles.

Kinetics of adsorption of the organic compounds by the SPME fiber were determined by measuring the amount of the probe compounds adsorbed at sampling times of 10 min, 30 min, 1 h, 2 h, 3 h, and 5 h. Each sample was analyzed three times. Linearity of the adsorption of the fiber was evaluated on samples spiked with four concentration levels of probe compounds in a range that included the concentrations values reported in Table 1. Each concentration level was analyzed three times. The adsorption of the compounds on the fiber obtained with and without vial agitation was also compared, using the automatic agitation of the sample vial during the SPME adsorption step provided by the autosampler to enhance the interaction between the fiber and the compounds in solution.

### SPME/GC-MS instrumentation and analytical parameters

Quantitative analyses of the probe compounds were performed with a gas chromatograph Agilent 7890A equipped with a mass spectrometer 5975C (Agilent, Palo Alto, CA, USA) and an autosampler Gerstel MPS configured for automated SPME analysis. The fiber was desorbed in splitless mode at 220 °C for 5 min in the injection port of the gas chromatograph equipped with a Siltek SPME liner 0.75 mm (id). Separation of the analytes was performed on a J&W HP-5MS capillary column, 30 m × 0.25 mm × 0.25 μm film thickness (Agilent, Palo Alto, CA, USA). The column oven was programmed as follows: initial temperature 40 °C for 3 min, ramped at 20 °C/min to 300 °C for 7 min. Carrier gas was helium at a flow rate of 1.5 mL/min.

### Quantification of the adsorption of probe compounds by NPs

The experimental design was aimed to the identification and quantification of the probe compounds adsorbed by the NPs with the optimization of the analytical parameters and the amount of probe compounds and NPs required for the adsorption test.

A preliminary screening of the probe compounds adsorbed on NPs surface was carried out with the compounds and concentrations reported in Table 1. Samples were maintained at room temperature overnight to reach adsorption equilibrium and the content of probe compounds that remained in solution was quantitatively determined by SPME/GC-MS (see “Sample preparation” section). The residual amount of the probe compound that remained in solution at the equilibrium was quantified using the control sample (“Quantification of the adsorption of probe compounds by SPME fiber” section) as external standard. The response factor was calculated as the ratio between the peak area and the concentration of the probe compound in the control. The amount of the probe compound adsorbed by NPs was calculated as the difference between the initial concentration of the probe compound and the amount that remained in solution at equilibrium. The analyses were carried out in triplicate with solutions containing single probe compounds. The calibration of the instrument using a single external calibration point (control sample) was used to simplify the analytical procedure as far as the linearity of the analytical response was verified over the whole concentration range of the probe compound.

Adsorption kinetic experiments were successively carried out to determine the equilibrium time for the adsorption of each compound by the SiO_2_ and Au NPs. The samples, prepared as described in “Quantification of the adsorption of probe compounds by SPME fiber” section, were analyzed in triplicate after 0, 0.5, 1, 2, 3, and 12 h of incubation at room temperature and the amount adsorbed at each incubation time was calculated. Found equilibrium time (12 h) was used for sample preparation in all further experiments. Usually, samples were left overnight before SPME/GC-MS analysis in order to achieve adsorption equilibrium.

Kinetic and linearity of adsorption of the probe compounds on SPME fiber is the second type of adsorption kinetic experiments in optimization process. It was performed on samples where equilibrium between probe compounds and NPs was already achieved. This parameter had to be optimized due to physical characteristics of SPME fiber and in order to obtain sensitive and accurate results.

### Calculation of the adsorption coefficient of the probe compounds

The amount of probe compounds adsorbed on the NPs surface calculated from the SPME/GC-MS method was used for calculation of the adsorption coefficients (k) of probe compounds expressed by the formula:

$$ k=\frac{V_0\left({C}_0-{C}_e\right)}{m{ c}_e} $$

where

*V*_*0*_: Volume of the sample
*c*_*0*_: Initial concentration of the probe compound in solution
*c*_*e*_: Concentration of the probe compound in solution at the equilibrium
*m*: Mass of the nanomaterial

The logarithm of the adsorption coefficient obtained (log k) is then calculated to be used in the calculation of nanodescriptors by means of multiple linear regression analysis [8, 9].

## Results and discussion

### Kinetic and linearity of adsorption of the probe compounds on SPME fiber

**Fig. 1:**
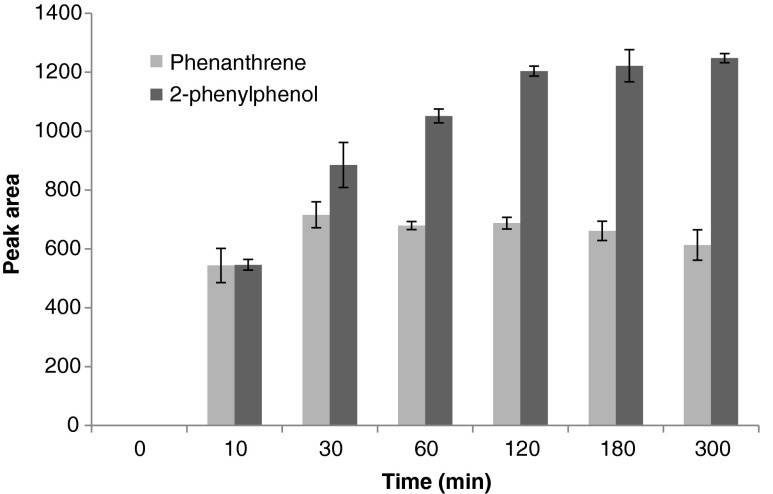
Adsorption kinetics of 2-phenylphenol (0.075 μg mL^−1^) and phenanthrene (0.080 μg mL^−1^) onto SPME fiber. Measurements were done in three repetitions after 10, 30, 60, 120, 180, and 300 min

Examples of the kinetic of adsorption of probe compounds on DVB/PDMS fiber are reported for 2-phenylphenol and phenanthrene (Fig. 1). The adsorption of 2-phenylphenol progressively increased with the time to reach the equilibrium of adsorption only after 2 h, while the adsorption of phenanthrene was already complete after 30 min. The time for complete adsorption of a compound by the fiber depends on its physicochemical properties and different compounds can have different equilibrium times. Although equilibrium state represents the optimal condition for quantification analysis with SPME [8, 9], correct results can be obtained also under non-equilibrium conditions [10]. This is particularly important for the sampling into the liquid phase where very long equilibrium times can excessively slow down the experimental work.

**Fig. 2:**
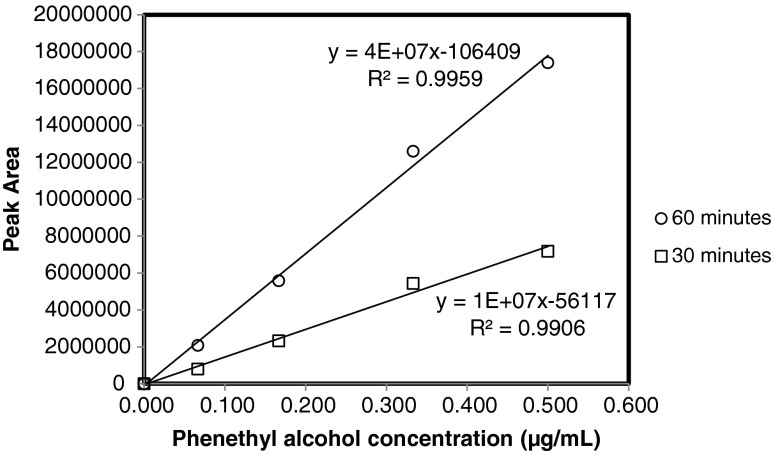
Calibration curves obtained for phenethyl alcohol at sampling time of 60 and 30 min. The samples were prepared at four different concentrations (0.067, 0.167, 0.333, 0.500 μg mL^−1^) of probe compound

**Fig. 3:**
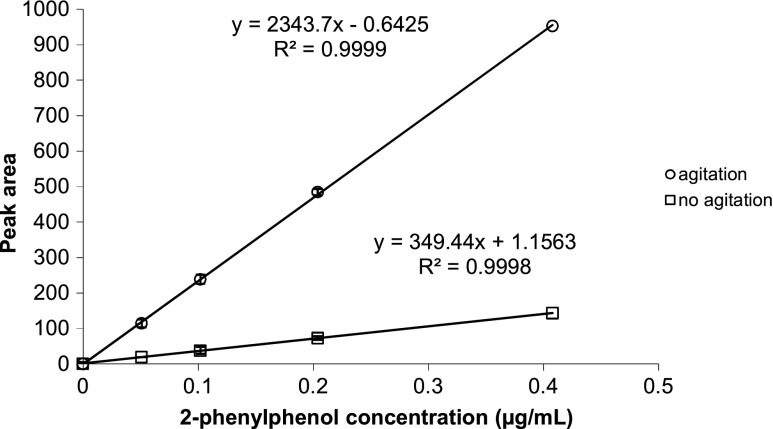
Calibration curves of 2-phenylphenol obtained with agitation of the sample and without agitation at an adsorption time of 30 min. The samples were prepared at four different concentrations (0.051, 0.102, 0.204, and 0.408 μg/mL) of probe compound. Measurements were done in three repetitions

The results of the linearity of the adsorption of the fiber obtained at 30 and 60 min of adsorption time are presented for phenethyl alcohol (Fig. 2). The linearity obtained at the different adsorption times were similar, indicating that a correct quantification of the compounds can be obtained also at nonequilibrium. Obviously, the lower amount of probe compound adsorbed at shorter times decreases the sensitivity of the analysis. On the basis of these results, an adsorption time of 30 min was considered as a suitable value to obtain an adequate analytical sensitivity at a reasonable analytical time and it was applied for all the next SPME quantification of probe compounds. The low volume of the liquid sample (200 μL) facilitated the use of shorter times, enhancing the interactions between the fiber and the compounds in solution. These interactions were further enhanced by the agitation of the sample during the fiber adsorption, which increased the amount of the adsorbed compound. The effect of the agitation of the liquid during the SPME adsorption on the analytical results is shown for the calibration curve for 2-phenylphenol at adsorption time of 30 min (Fig. 3). The linearity of the calibration curves obtained with and without agitation was comparable but the curve obtained with the agitation of the liquid sample showed much higher peak area values. The maximum values of relative standard deviations for the three repetitions of each concentration were 4.7 and 5.6 % for the sampling with and without agitation, respectively.

### Adsorption of the probe compounds on NPs

A preliminary screening carried out to select the substances adsorbed by the two NPs pointed out differences between the activity of SiO_2_ NPs and Au NPs. Since the focus of our study was optimization of the SPME-GC/MS method, we used both the NPs for the experimental study. The application of the method to different kinds and sizes of NPs will be considered in future studies.

Our results indicated that both NPs were in the weak adsorption zone. SiO_2_ NPs, in particular, showed a very low free-surface activity with significant adsorption of only three of the tested compounds (Table 2). The results are consistent with previous studies on SiO_2_ NPs that revealed low surface activity for this NPs [8, 9]. Au NPs adsorbed more compounds and at higher amounts compared to SiO_2_ NPs (Table 2). A different behavior between the two NPs was expected because of their different surface chemistry. For this reason, different sets of probe compounds may be needed for accurate determination of the nanodescriptors for different NPs.

**Table 2:** Probe compounds adsorbed on Au (15 nm) and SiO_2_ (50 nm)

Probe compound	Au (15 nm)		SiO_2_ (50 nm)	
	ng mL^−1^	ng adsorbed	ng mL^−1^	ng adsorbed
Ethylbenzene			23.4	1.5
Propylbenzene			25.5	2.1
4-Chlorotoluene			29.9	2.1
2-Phenylphenol	408	41		
1-Methylnaphthalene	97	10		
Biphenyl	77	5		
Phenanthrene	545	42		
Azobenzene	255	27		
Pyrene	524	36		
1-Ethylnaphthalene	224	29		
Naphtalene	195	4		
3,5-Dimethylphenol	390	19		

The screening test revealed also unexpected reactions in sample solutions of some probe compounds in the presence of Au NPs. In fact, the decreasing of the amount of some compounds was accompanied by the appearance of new chromatographic peaks. The effect may be explained by chemical reactions in sample solutions catalyzed by NPs that led to the formation of new substances. In particular, benzyl alcohol and 3-methylbenzyl alcohol were converted to methyl esters of benzoic and methyl benzoic acid, respectively, and iodobenzene was converted to biphenyl. Since the effect was not observed in the control samples (without NPs) and the liquid injection of hexane extracts of the samples confirmed the presence of the new compounds, a catalytic effect of these reactions by NPs is a plausible explanation.

The adsorption of NPs by the SPME fiber was also investigated to exclude their presence on the fiber surface that could affect the adsorption behavior of the fiber. The amount of nanoparticles adsorbed on the fiber surface was analyzed using X-ray photoelectron spectroscopy (XPS) after the sample adsorption and after a long-term use. The results of XPS analysis indicated the presence of adsorbed nanoparticles in very low amount with a slight increase with the time of use of the fiber, which excluded a significant influence on the adsorption efficiency of the fiber.

### Kinetic of adsorption of the probe compounds on Au NPs

**Fig. 4:**
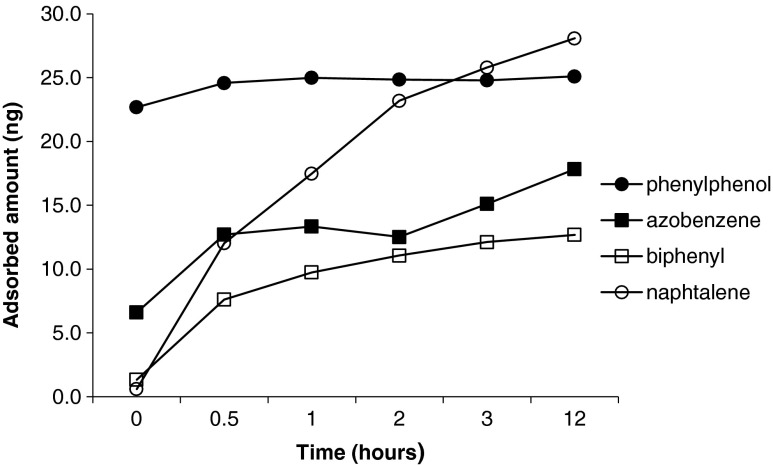
Kinetic of adsorption for 2-phenylphenol, naphthalene, azobenzene, and biphenyl on by Au NPs (15 nm) incubated in different times (0.5, 1, 2, 3, and 12 h). The samples were prepared at initial concentrations of 0.075 μg mL^−1^ for 2-phenylphenol, 0.018 μg mL^−1^ for naphthalene, 0.105 μg mL^−1^ for azobenzene, and 0.018 μg mL^−1^ for biphenyl

The adsorption kinetics of 2-phenylphenol, naphthalene, azobenzene, and biphenyl on Au NPs are shown as examples of the probe compounds adsorption kinetic of NPs (Fig. 4). The values at time 0 necessarily included the adsorption obtained during the 30-min fiber sampling for the SPME analysis. The graph shows major differences in the behavior of the probe compounds, which exhibited high variations in their adsorption rates. The examples presented included compounds, like 2-phenylphenol, that were very quickly adsorbed by the NPs and reached the equilibrium already after 30 min and compounds, such as naphthalene, with very slow adsorption rates whose equilibrium time exceeded 12 h. These findings are in contradiction with other studies, which found that an average equilibrium time of 5 h should be taken for the same probe compounds [8, 9]. Based on these results, we decided to apply overnight incubation as an average time to all the successive adsorption experiments to make sure that we always worked within the equilibrium regime.

### Optimization of NPs concentration

**Fig. 5:**
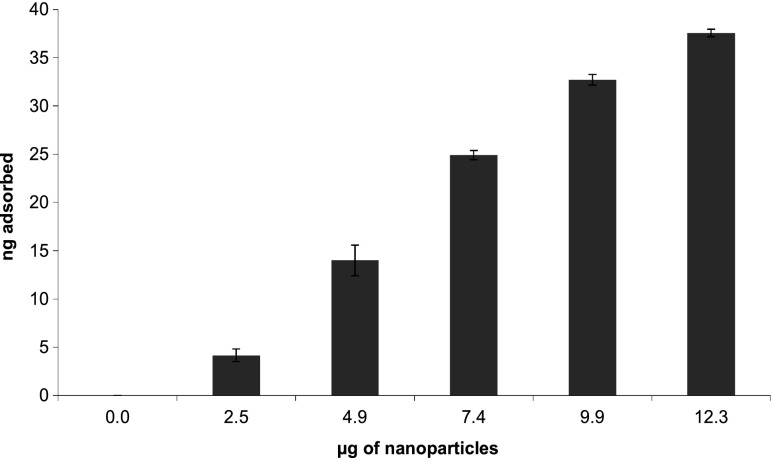
Adsorption of 2-phenylphenol by different amounts of Au nanoparticles. The samples were prepared with various amounts of Au (2.5, 4.9, 7.4, 9.9, and 12.3 μg), and 0.075 μg mL^−1^ of probe compound and incubated overnight in order to achieve NPs-probe compound adsorption equilibrium. Measurements were done in three repetitions

The adsorption of 2-phenylphenol on 15-nm Au NPs with different concentrations is reported as example of the influence of NPs concentration on the adsorption of probe compounds (Fig. 5). The results showed that the amount of probe compound adsorption was proportional to the amount of NPs, with a minimum adsorption of 4.2 ng and a maximum of 37.6 ng that corresponded to an almost complete adsorption of the probe compound initially present in solution (40 ng). The optimization of the ratio between the amount of NPs and probe compounds represented an important step for a correct evaluation of the adsorption properties of NPs. The experiment permitted to find the minimum concentration of probe compound that could be added for a specific NPs concentration, without a complete adsorption on the NPs surface. This was particularly important in the procedure for the characterization of adsorption properties of the NPs that require the addition of multiple probe compounds to evaluate their competitive adsorptions on the NPs surfaces under evaluation [8, 9]. Indeed, it is advisable to reduce as much as possible the concentrations of the probe compounds in the adsorption experiments in order to avoid the occurrence of multiple layer adsorptions on the particles surface, which could led to an overestimation of the adsorbed amount [8, 9]. The information derived from the comparison of the results obtained for the single probe compounds and for the specified NPs can offer an overview of NPs surface activity that facilitate the choice of initial concentrations of probe compounds and NPs. Moreover, the concentration of the probe compounds in solution is a critical parameter also for the SPME analysis since the simultaneous adsorption of multiple components in excessive amounts could cause saturation of the fiber which would lead to the consequent uncorrected quantification of the adsorption.

### Calculation of adsorption coefficients of the probe compounds

The log adsorption coefficients values (log k) obtained for 1-methylnaphtalene and biphenyl are presented as examples of the application of the optimized SPME/GC-MS method in the NPs surface characterization (Table 3.). The values were obtained with the addition of single compounds in solution with Au NPs (15 nm). The results show a good repeatability with a relative standard deviation of 5 % for the two probe compounds. The calculated values were compared with predicted log k values found in literature; obtained using molecular descriptors developed in quantitative structure–activity relationship (QSAR) studies [19]. The calculated mean values for adsorption coefficients for 1-methylnaphthalene and biphenyl were lower than predicted values but without an excessive deviation. These first results indicated that the SPME/GC-MS procedure can be successfully applied to the quantification of adsorption coefficients of NPs, although further studies are required for an exhaustive evaluation of its variability. In particular, the optimization of experimental parameters will aim at the quantification of the adsorption by NPs exposed to multiple probe compounds to evaluate the result of their competitive adsorption for a more comprehensive description of the adsorption affinity of the NPs.

**Table 3:** Logarithm of adsorption coefficients values for 1-methylnaphthalene and biphenyl in presence of Au NPs (15 nm), expressed as mean values of 3 repetitions

Probe compound	Literature value	Calculated value
1-Methylnaphtalene	4.89	4.66 ± 0.23
Biphenyl	5.18	4.44 ± 0.26

## Conclusions

In this study, we have shown detailed optimization process of a SPME/GC-MS method used for characterization of NPs surface reactivity. The SPME analytical procedure applied permitted a fast and reliable quantification of the probe compounds without affecting the interactions probe compounds/nanomaterials.

A preliminary screening of the adsorption performed with 33 probe compounds and 2 NPs (Au, 15 nm, and SiO_2_, 50 nm) indicated that both nanomaterials are in weak adsorption zone, SiO_2_ NPs in particular, with adsorption equilibrium times ranging from 30 min to 12 h. It is assumed that NPs could catalyze additional chemical reactions of some probe compounds.

Adsorption experiments with different amounts of probe compounds and nanoparticles allowed establishing the minimum concentration of each probe compound required to avoid its complete adsorption by a specific NPs concentration in order to perform correct quantification of the nanomaterial surface activity, especially when simultaneous adsorption of multiple components is carried out.

The log k values calculated for 1-methylnaphtalene and biphenyl in solution with Au NPs (15 nm) showed good analytical repeatability (RSD 5 %) and satisfactory correspondence with predicted log k values found in literature, obtained using molecular descriptors developed in quantitative structure–activity relationship (QSAR) studies [19].

This paper showed that optimized SPME/GC-MS procedure can be successfully applied to the quantification of adsorption coefficients of NPs. Further studies are required for an exhaustive evaluation of its variability, in particular for multiple probe compounds competitive adsorption and in the presence of proteins and peptides.
